# Supplementary material for: Impulsive Personality Traits Predicted Weight Loss in Individuals with Type 2 Diabetes after 3 Years of Lifestyle Interventions
Source: J Clin Med. 2022 Jun 16;11(12):3476. doi: 10.3390/jcm11123476 (PMC9224680; doi:10.3390/jcm11123476)

Journal of
Clinical Medicine

Table S1 (supplementary). Cross-sectional association between impulsivity levels (UPPS-P scores) with insulin related metabolic disorders in TD2+ patients at baseline: partial correlations adjusted by age, sex and education (analysis within the TD2+ group, *n*=139).

UPPS-P scores	HOMA	Glucose	HbA_{1c}
Lack of premeditation	0.007	0.053	−0.034
Lack of perseverance	0.066	−0.018	−0.042
Sensation seeking	0.003	−0.087	−0.098
Positive urgency	−0.022	−0.095	−0.023
Negative urgency	0.009	−0.061	−0.012

Note. TD2(−): diabetes absent. TD2(+): diabetes present. HOMA: homeostatic model assessment. HbA_{1c}: glycated hemoglobin. †Bold: effect size into the range mild-moderate to high-large.

Figure S1. Flowchart for the sampling

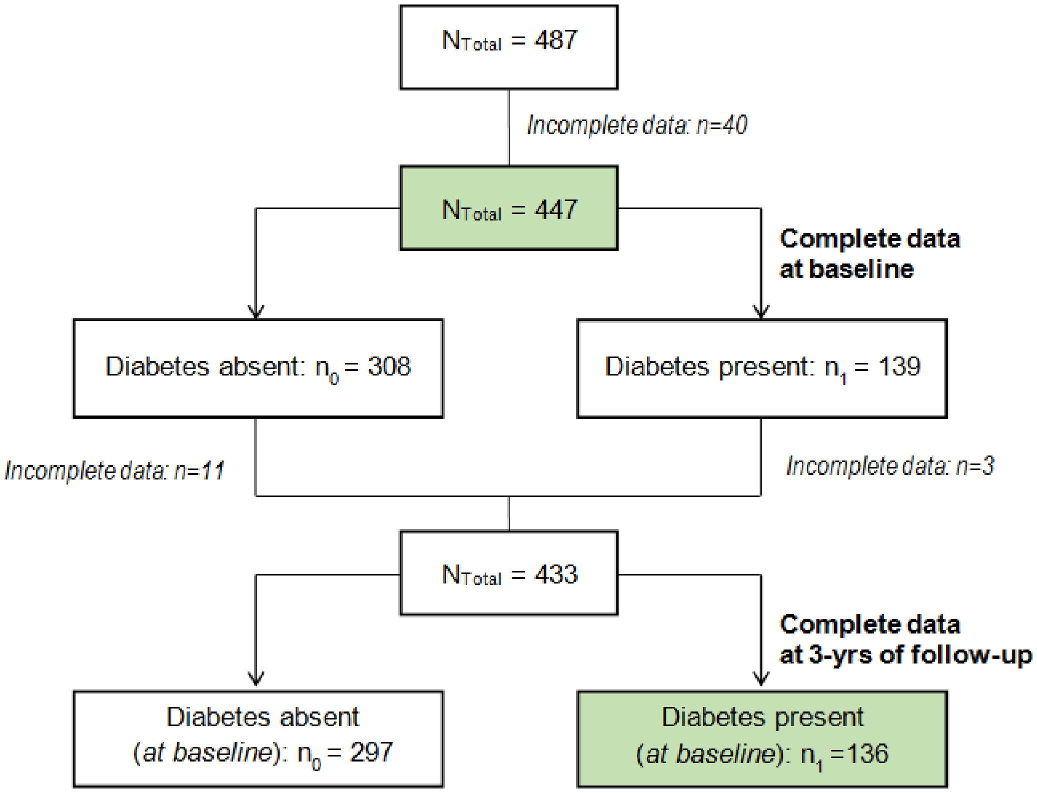

Supplement: Supplementary file 1 [file jcm-11-03476-s001.zip › jcm-1727352-supplementary.pdf]
